# Performance of lung cancer screening with low‐dose CT in Gejiu, Yunnan: A population‐based, screening cohort study

**DOI:** 10.1111/1759-7714.13379

**Published:** 2020-03-20

**Authors:** Meng‐Na Wei, Zheng Su, Jian‐Ning Wang, Maria J. Gonzalez Mendez, Xiao‐Yun Yu, Hao Liang, Qing‐Hua Zhou, Ya‐Guang Fan, You‐Lin Qiao

**Affiliations:** ^1^ Department of Cancer Epidemiology, National Cancer Center/National Clinical Research Center for Cancer/Cancer Hospital Chinese Academy of Medical Sciences and Peking Union Medical College Beijing China; ^2^ Office of Gejiu Municipal Leading Group for Cancer Prevention and Control Gejiu China; ^3^ School of Public Health Dalian Medical University Dalian China; ^4^ Lung Cancer Center/Lung Cancer Institute West China Hospital, Sichuan University Chengdu China; ^5^ Tianjin Key Laboratory of Lung Cancer Metastasis and Tumor Microenvironment, Tianjin Lung Cancer Institute Tianjin Medical University General Hospital Tianjin China

**Keywords:** Cohort study, LDCT, lung cancer, screening

## Abstract

**Background:**

The performance of lung cancer screening with low‐dose computed tomography (CT) (LDCT) in China is uncertain. This study aimed to evaluate the performance of LDCT lung cancer screening in the Chinese setting.

**Methods:**

In 2014, a screening cohort of lung cancer with LDCT was established in Gejiu, Yunnan Province, a screening center of the Lung Cancer Screening Program in Rural China (LungSPRC). Participants received a baseline screening and four rounds of annual screening with LDCT in two local hospitals until June 2019. We analyzed the rates of participation, detection, early detection, and the clinical characteristics of lung cancer.

**Results:**

A total of 2006 participants had complete baseline screening results with a compliance rate of 98.4%. Of these, 1411 were high‐risk and 558 were nonhigh‐risk participants. During this period, 40 lung cancer cases were confirmed, of these, 35 were screen‐detected, four were post‐screening and one was an interval case. The positive rate of baseline and annual screening was 9.7% and 9.0%, while the lung cancer detection rate was 0.4% and 0.6%, respectively. The proportion of early lung cancer increased from 37.5% in T0 to 75.0% in T4. Adenocarcinoma was the most common histological subtype. Lung cancer incidence according to the criteria of LungSPRC and National Lung Cancer Screening Trial (NLST) was 513.31 and 877.41 per 100 000 person‐years, respectively.

**Conclusions:**

The program of lung cancer screening with LDCT showed a successful performance in Gejiu, Yunnan. However, further studies are warranted to refine a high‐risk population who will benefit most from LDCT screening and reduce the high false positive results.

**Key points:**

This study reports the results of lung cancer screening with LDCT in Gejiu, Yunnan, a high‐risk area of lung cancer, and it demonstrates that lung cancer screening with LDCT is effective in detecting early‐stage lung cancer. Our program provides an opportunity to explore the performance of LDCT lung cancer screening in the Chinese context.

## Introduction

Lung cancer is the most common cancer worldwide, including China.^1^, ^2^, ^3^, ^4^ In 2015, it was estimated that 733 300 lung cancer cases were diagnosed, and 610 200 deaths occurred due to lung cancer in China.^4^ In China, the age‐standardized five‐year relative survival rate (2012–2015) of lung cancer was reported to be 19.7%.^5^ Fortunately, lung cancer diagnosed at an early stage has a better prognosis, with a five‐year survival rate of 77%–92%.^6^ This information underlines the need for lung cancer screening in China. In 2011, the NLST demonstrated a 20% reduction in lung cancer‐related mortality with low‐dose computed tomography (CT) (LDCT) screening.^7^ Since 2013, lung cancer screening has been recommended by the United States Preventive Services Task Force.^8^ Based on this context, some programs have been carried out to assess the performance of lung cancer screening with LDCT in China.^9^, ^10^, ^11^, ^12^, ^13^ However, the performance of population‐based lung cancer screening with LDCT in China is uncertain.

Since 2009, the Lung Cancer Screening Program in Rural China (LungSPRC) has been included in the cancer early detection and treatment program, a special funded program supported by the Chinese Central Government Public Health Special Subsidy.^9^ Gejiu, a small city in Yunnan Province and the site of the largest Asian tin industry, has been reported to have the highest male lung cancer mortality among all Chinese areas, as described in a 1973 nationwide survey.^14^ Great efforts have been made in Gejiu city to move forward in cancer surveillance and control, but lung cancer is still the first leading cause of cancer‐related mortality there.^15^ In 2014, Gejiu was selected as a screening center of LungSPRC. The present study describes the results of the screening program in Gejiu city.

## Methods

### Study design

This is an ongoing, lung cancer screening cohort study with LDCT. Participants were enrolled based on a cluster sampling design. It has been approved by the Cancer Foundation of China Institutional Review Board. A written informed consent was obtained from all participants voluntarily.

### Study population

In 2014, residents from Retirement Management Centre of Yunnan Tin Corporation and four communities (Baohua, Shanghe, Yanpeng and Xinzhai) were invited to participate in lung cancer screening with LDCT. We finally recruited 2006 (59.5% men and 40.5% women) participants with complete baseline LDCT screening results to establish a lung cancer screening cohort in Gejiu city. The recruited participants were classified as high‐risk population and nonhigh‐risk population according to the risk of lung cancer. The factors for considering an individual as high‐risk were as follows: (i) Participants were 40–74‐years‐old with occupational exposure, or 50–74‐years‐old without occupational exposure; (ii) at least 20 pack‐years smoking history, and, if former smokers, had quit within the previous five years; (iii) having a history of 10 or more years of underground mining and/or smelting experience. Participants who satisfied the criteria (i) and (ii), or (i) and (iii), or (i) (ii) (iii) were considered as high‐risk. If the participants did not meet any of the previous mentioned criteria, or they met only one, they were classified as nonhigh risk. Individuals who had one of the following criteria were excluded: (i) having a history of any cancer within the last five years (except for nonmelanoma skin cancer, cervical cancer in situ and localized prostate cancer); (ii) unable to tolerate possible lung cancer resection; and (iii) with life threatening diseases. We performed a subgroup analysis among those participants who satisfied the LungSPRC criteria and divided them into three groups. Those who were aged 50–74 years and had at least 20 pack‐years smoking history, but without occupational exposure. were classified as group 1. Group 2 included participants who were aged 40–74 years and had a history of smoking and underground mining, and/or smelting. Group 3 included participants who were aged 40–74 years and had a history of 10 or more years of underground mining and/or smelting experience, but without smoking history. We also performed a subgroup analysis based on the occupational history of the participants. If the subjects had experience of underground mining and/or smelting, they were classified as tin miners, otherwise as nontin miners.

### Baseline survey

At the time of recruitment, all participants filled in a questionnaire with the assistance of well‐trained investigators. The detailed information included personal characteristics, lifestyle, previous medical history, occupational exposure and family history of cancer. Pack‐year was calculated by multiplying the number of packs of cigarettes smoked per day by the number of years the person had been smoking (for water pipes and Chinese long‐stem pipes, 1.25 g pipes = 1 cigarette). The participant was considered to have a prior lung disease if he or she had certain nonmalignant lung diseases, such as asthma, chronic bronchitis, emphysema, silicosis, tuberculosis (TB) or chronic obstructive pulmonary disease (COPD) at the time of enrollment.

### LDCT screening

After the survey questionnaire, all participants were invited to undergo LDCT baseline screening (T0) in Honghe Prefecture third people's hospital and Gejiu people's hospital by experienced radiologists and radiological technologists. Four rounds of incidence or annual screening (T1, T2, T3 and T4) took place after the baseline screening at one‐year intervals until June of 2019. LDCT screening was performed following a standard protocol developed by related experts from different fields.^9^ The CT scan was completed at the end of an inhalation within a single breath‐hold through the whole lung. All images were collected with the following examination parameters^9^: 120 kVp; 30 mAs; 350 mm scan length; 3.7 seconds scan time; reconstruction slice thicknesses contiguous 0.625–1.25 mm (slice interval of 0); if the conditions were not adequate, a contiguous 5 mm thick reconstruction slice thickness could be adopted. Images were interpreted first by two senior radiologists from local hospitals and then a provincial expert panel resolved any differences.

In the baseline screening, participants were considered as positive with solid or part‐solid nodules ≥5 mm in diameter, or nonsolid nodules ≥8 mm in diameter, or airway lesion, nodules and masses suspicious for lung cancer. At the annual screening, any new noncalcified nodules or new airway lesion was considered as positive. In addition, enlarged nodules or nodules with an increase of solid components were also considered as positive. The anatomical location (lobe), longest axial, perpendicular diameters, margin characteristics and type of nodules were recorded for all positive nodules. Histopathological type and tumor stage were assessed using the 2015 World Health Organization (WHO) classification of tumors for the lung, pleura, thymus, and heart and the eighth edition of the TNM Classification for Lung Cancer.^16^, ^17^

### Follow‐up of participants

The detailed management of the baseline and annual LDCT screening results was based on the published literature.^9^ If participants were diagnosed with lung cancer, they did not receive any further LDCT screening. Related diagnostic procedures, anatomical location, histological subtype, tumor stage and initial treatment were documented by a certified medical record. Those who did not participate in the annual LDCT screening were followed up via Gejiu Cancer Registry and telephone call to attain the health outcome. A lung cancer with a positive LDCT scan and diagnosed within 12 months, or before the next round of screening, was classified as a screen‐detected cancer. An interval lung cancer was a cancer with a negative LDCT scan and diagnosed within 12 months or before the next screening. A participant who did not participate in the next continuous screening, or any other screening after the previous screening round, and was diagnosed with lung cancer 12 months later, was classified as a post‐screening lung cancer. According to the screening tasks issued by the Ministry of Health, 1000 individuals who completed baseline LDCT screening were called back for annual screening every year. Stage I lung cancer and carcinoma in situ were defined as early‐stage cancers.

### Statistical analysis

The participation rate, detection rate, early detection rate, and the clinical characteristics of lung cancer were reported to evaluate the program performance. The detection rate was defined as the number of screen‐detected lung cancer among the screened participants. The early detection rate was the number of early‐stage lung cancer to the screen‐detected lung cancer. The person‐years measurement that takes into account the number of people in the study and the amount of time each person spent in the study, was calculated from the baseline time of LDCT screening to the date of lung cancer diagnosis, death or censoring (whichever came first). In this study, we only reported positive rate of noncalcified nodules in T0, T1, T2 and T3 screening round.

Categorical variables, including gender (male or female), age at baseline (40‐, 50‐ or 60‐ years old), education (≤ primary education, middle school, or college or higher), cigarette smoking (no, current or former), pack‐years (0, <20 or ≥ 20), arsenic exposure (0, < 10 or ≥ 10 years), radon exposure (0, < 10 or ≥ 10 years), prior lung disease (no or yes), family history of cancer (no or yes), tin miner (no or yes), were presented as number and frequency. The differences were compared by Chi‐square test, or Fisher's exact test, as appropriate. *P* < 0.05 was considered to indicate statistical significance using the two‐tailed test. IBM SPSS Statistics 23 software was used for the final statistical analysis.

## Results

### Baseline characteristics of participants

**Figure 1:**
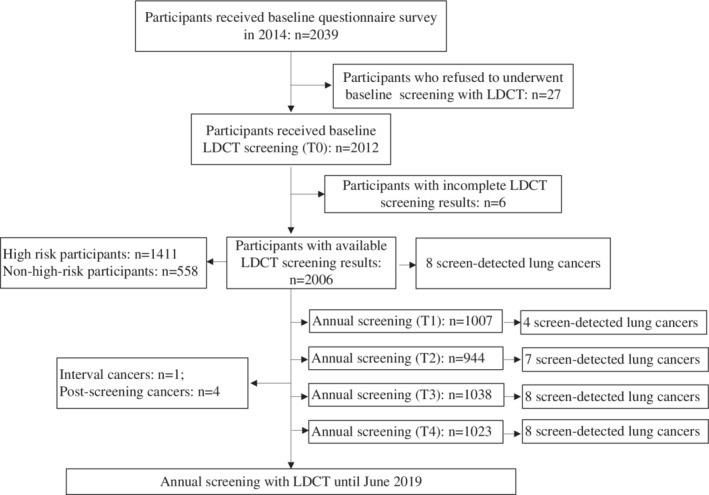
Flowchart of lung cancer screening with LDCT of participants. T0, baseline screening; T1, the first round of annual screening; T2, the second round of annual screening; T3, the third round of annual screening; T4, the fourth round of annual screening.

Figure 1 shows the flowchart of lung cancer screening with LDCT in Gejiu, Yunnan Province. From June 2014 to December 2014, a total of 2039 subjects filled in a baseline questionnaire; 27 subjects refused to undergo baseline LDCT screening, and six participants had incomplete screening results. A total of 2006 participants had available baseline CT results with a compliance rate of 98.4% (2006 of 2039). According to the LungSPRC protocol, 1411 participants were classified as high‐risk population and 558 as nonhigh‐risk population after excluding those aged more than 74‐years‐old (*n* = 6), and those lacking explicit year of smelting experience (*n* = 31). The baseline characteristics of included participants are shown in Table 1. Compared with the nonhigh‐risk population, the high‐risk population tended to be older, had more males (72.6% vs. 28.0%), a higher education level (67.8% vs. 58.8% ≥ middle school), more current and former smokers (62.8% vs. 17.9%), a higher pack‐years index (47.6% vs. 3.2% ≥20 pack‐years), higher exposure to arsenic (10.7% vs. 0.0% ≥10 years) and radon (76.7% vs. 0.0% ≥10 years), a greater proportion of family history of cancer (30.3% vs. 25.3%), and more tin miners (89.2% vs. 5.4%) (all *P* < 0.05).

**Table 1:** Baseline characteristics of participants

Characteristics n (%)	Screened participants *N* = 2006	High‐risk *N* = 1411	Nonhigh‐risk *N* = 558	*P*‐value
Gender				<0.0001
Male	1193 (59.5)	1024 (72.6)	156 (28.0)	
Female	813 (40.5)	387 (27.4)	402 (72.0)	
Age (years)				<0.0001
40‐	202 (10.1)	83 (5.9)	119 (21.3)	
50‐	990 (49.4)	756 (53.6)	214 (38.4)	
60‐	814 (40.6)	572 (40.5)	225 (40.3)	
Education				0.0007
≤Primary education	694 (34.6)	454 (32.2)	230 (41.2)	
Middle school	1272 (63.4)	929 (65.8)	317 (56.8)	
College or higher	40 (19.9)	28 (2.0)	11 (2.0)	
Cigarette smoking				<0.0001
No	1003 (50.0)	525 (37.2)	453 (82.1)	
Current	796 (39.7)	706 (50.0)	80 (14.3)	
Former	207 (10.3)	180 (12.8)	25(4.5)	
Pack‐year				<0.0001
0	1003 (50.0)	525 (37.2)	453 (81.2)	
<20	306 (15.3)	214 (15.2)	87 (15.6)	
≥20	695 (34.6)	672 (47.6)	18 (3.2)	
Arsenic (years)				<0.0001
0	1809 (90.2)	1253 (88.8)	550 (98.6)	
<10	15 (0.7)	7 (0.5)	8 (1.4)	
≥10	182 (9.1)	151 (10.7)	0 (0.0)	
Radon (years)				<0.0001
0	860 (42.9)	291(20.6)	534(95.7)	
<10	62 (3.1)	38 (2.7)	24 (4.3)	
≥10	1084 (54.0)	1082 (76.7)	0 (0.0)	
Prior lung disease				0.77
No	1880 (93.7)	1320 (93.6)	524 (93.9)	
Yes	126 (6.3)	91 (6.4)	34 (6.1)	
Family history of cancer				0.028
No	1424 (71.0)	984 (69.7)	417 (74.7)	
Yes	582 (29.0)	427 (30.3)	141 (25.3)	
Miner				<0.0001
No	685 (34.1)	153 (10.8)	528 (94.6)	
Yes	1321 (65.9)	1258 (89.2)	30 (5.4)	

### Nodules detected at baseline and annual screening

Out of 2006 participants, 1696 (84.5%) underwent at least one annual screening (85.4% of high‐risk participants and 83.2% of nonhigh‐risk participants). A total of 272 noncalcified positive nodules were found in 195 participants in the baseline screening (T0); of these, 235 nodules were solid or part‐solid nodules with a diameter ≥ 5 mm, 14 nodules were nonsolid nodules with a diameter ≥ 8 mm, and 23 nodules had a diameter ≥ 15 mm. The positive rates of baseline and annual screening (T1‐T3) were 9.7% and 9.0%, respectively, while the lung cancer detection rate of baseline and annual screening was 0.4% and 0.6% (T1–T3), respectively **(**Table 2). In the first four rounds, 19.4% (389 of 2006) of participants had at least one positive screening result; however, only 8.7% (34 of 389) were true positives.

**Table 2:** Positive rate and yield of lung cancer

	Round	Screened participants (N)	Positive rate n (%)	Detection of lung cancer n (%)
Baseline	T0	2006	195 (9.7)	8 (0.4)
Annual	T1	1007	133 (13.2)	4 (0.4)
	T2	944	47 (5.0)	7 (0.7)
	T3	1038	88 (8.5)	8 (0.8)
	T1–T3	2989	268 (9.0)	19 (0.6)

T0, baseline screening; T1, the first round of annual screening; T2, the second round of annual screening; T3, the third round of annual screening.

### Characteristics of lung cancer detected in baseline and annual screening

After baseline screening (T0) and four rounds of annual screening (T1, T2, T3, and T4), 35 screened participants were confirmed as screen‐detected lung cancers (30 in the high‐risk population and five in the nonhigh‐risk population). On the other hand, four participants were diagnosed as post‐screening lung cancers, and one participant was diagnosed as an interval cancer. Adenocarcinoma was the most common histological subtype in T0 (50.0%), T1 (50.0%), T3 (71.4%) and T4 (71.4%) except for T2 (20.0%). Among screen‐detected lung cancers, the proportion of early‐stage lung cancer (stage I and carcinoma in situ) increased from 37.5% in T0 to 75.0% in T4 round (Table 3).

**Table 3:** Characteristics of screen‐detected lung cancer within baseline and annual screening

		Annual n (%)				
Characteristics	T0 n (%)	T1	T2	T3	T4	Total
Histology						
Adenocarcinoma	4 (50.0)	2 (50.0)	1 (20.0)	5 (71.4)	5 (71.4)	13 (56.5)
Squamous	4 (50.0)	1 (25.0)	4 (80.0)	2 (28.6)	0 (0.0)	7 (30.4)
Other	0 (0.0)	1 (25.0)	0 (0.0)	0 (0.0)	2 (28.6)	3 (13.0)
Stage						
I and carcinoma in situ	3 (37.5)	3 (75.0)	2 (40.0)	5 (71.4)	6 (75.0)	16 (66.7)
II	3 (37.5)	1 (25.0)	0 (0.0)	0 (0.0)	0 (0.0)	1 (4.2)
III–IV	2 (25.0)	0 (0.0)	3 (60.0)	2 (28.6)	2 (25.0)	7 (29.2)

In T2, two lung cancer cases without stage and histological subtype were not shown; In T3, one lung case without stage and histological subtype was not shown; In T4, one lung case without histological subtype was not shown.

T0, baseline screening; T1, the first round of annual screening; T2, the second round of annual screening; T3, the third round of annual screening; T4, the fourth round of annual screening.

### Incidence of lung cancer according to different characteristics

The incidence of lung cancer was 417.93 per 100 000 person‐years for all participants. According to the LungSPRC protocol, participants who satisfied the criteria of high‐risk population had a higher incidence than those that did not meet the criteria (513.31 vs. 170.70 per 100 000 person‐years). Of those high‐risk participants, the subgroup analysis showed that group 1 (760.58 per 100 000 person‐years) had the highest incidence of lung cancer, followed by group 2 (683.25 per 100 000 person‐years) and group 3 (198.64 per 100 000 person‐years). Tin miners had a higher lung cancer incidence than nontin miners (468.85 vs. 308.49 per 100 000 person‐years). The stratified analysis by smoking among tin miners showed that tin miners who smoke had a higher lung cancer incidence (671.04 per 100 000 person‐years) than those who did not smoke (191.66 per 100 000 person‐years). Applying the NLST criteria for inclusion of the high‐risk population yielded a lung cancer incidence of 877.41 per 100 000 person‐years. Participants with a positive result had a higher risk of lung cancer than those with a negative result (1025.90 vs. 259.27 per 100 000 person‐years) (Table 4).

**Table 4:** Lung cancer incidence according to baseline characteristics (per 10^5^ person‐year)

Characteristics of participants	Number of participants	Person‐year	Lung cancer	Lung cancer incidence
All	1969	9571.06	40	417.93
LungSPRC criteria				
Yes	1411	6818.54	35	513.31
Subgroup analysis				
Group 1	153	788.87	6	760.58
Group 2	733	3512.62	24	683.25
Group 3	525	2517.06	5	198.64
No	558	2932.52	5	170.70
Miner				
Yes	1288	6185.33	29	468.85
Subgroup analysis (smoking)				
Yes	745	3576.53	24	671.04
No	543	2608.80	5	191.66
No	681	3565. 73	11	308.49
NLST criteria				
Yes	491	2393.40	21	877.41
No	1478	7357.66	19	258.23
Baseline outcome				
Positive	185	877.28	9	1025.90
Negative	1776	8871.03	23	259.27

Group 1, participants aged 50–74 years, with at least 20 pack‐years smoking history, without occupational exposure; Group 2, participants aged 40–74 years, with a history of smoking and underground mining and/or smelting; Group 3, participants aged 40–74 years, with a history of 10 or more years of underground mining and/or smelting experience, without smoking history.

LungSPRC, Lung Cancer Screening Program in Rural China; NLST, National Lung Cancer Screening Trial.

## Discussion

Due to the severe impact of lung cancer on public health, efforts to prevent and control lung cancer have been ongoing for many decades. Randomized controlled trials focusing on chest radiography and sputum cytology have detected several cases of early‐stage lung cancer, but have not reduced lung cancer mortality.^18^, ^19^ In 2011, the NLST demonstrated a lung cancer mortality reduction of 20% via LDCT screening,^7^ which suggests that LDCT may be a promising tool for the early detection of lung cancer and for reducing mortality in high‐risk asymptomatic individuals. Currently, many lung cancer screening programs with LDCT have been initiated in China. However, many questions remain unanswered.

In our study, the participants' adherence to LDCT screening was high, which demonstrates that lung cancer screening with LDCT is accepted in Gejiu city. At the baseline screening, the positive rate was 9.7% for all participants and 11.3% for the high risk population, which was similar to the results in Shanghai (14.56%) reported by Li *et al*. 20 but lower than another study performed in Shanghai (29.89%),^11^ NELSON^21^ (20.8%), Henschke *et al*.^22^ (23.0%), the National Lung Screening Trial (NLST) *et al*.^23^ (27.3%) and ITALUNG^24^ (30.3%). One possible reason to explain this discrepancy may be the differences in the criteria defining a high‐risk population. In the NLST,^25^ the subjects who were aged between 50–74‐years‐old, had a smoking index ≥30 pack‐years, or cessation years <15, and considered as high‐risk subjects for lung cancer. In this study, inclusion criteria for high‐risk groups were lower than those for NLST. The smoking index (≥20 pack‐years) in our study was lower than NLST (≥30 pack‐years), and subjects in our study were younger (40.6% participants aged ≥60 years old) than NLST (57.1% participants aged ≥60 years old).^7^ Different definition of positive results could be another important reason.^24^, ^25^ In the NLST, noncalcified nodules ≥4 mm were defined as a positive result.^25^ The present study defined solid or part‐solid nodules ≥5 mm, nonsolid nodules ≥8 mm, airway lesion, nodules and masses suspicious for lung cancer as a positive result. The positive result criteria are higher than that of the NLST. The equipment used for LDCT screening, and radiological experts' experience in different regions may also have contributed to this discrepancy.

Previous studies have determined that new solid nodules detected at the incidence screening might have a higher probability of lung cancer than nodules detected in baseline.^26^, ^27^ In this study, the positive rate in annual screening was lower than the baseline, while the detection rate of lung cancer in annual screening (T1–T3) was higher than the baseline. The results of our study substantiate the findings of previous studies. Only one interval lung cancer was found during the study period, which supports the function of LDCT screening in detecting lung cancer. Among four post‐screening lung cancers cases, two of four refused clinical intervention or further screening because of economic difficulties, and the remaining two cases did not undergo continuous annual screening because of limited screening tasks. Increasing screening tasks in Gejiu city may reasonably solve this problem.

Adenocarcinoma was the most common histological subtype both in baseline and annual screening followed by squamous cell carcinoma, which was consistent with previous studies.^23^, ^24^, ^28^ The proportion of early‐stage lung cancer (stage I and carcinoma in situ) at the annual screening (66.7%) was higher than the baseline screening (37.5%), which indicates that LDCT screening is effective in detecting early‐stage lung cancer. The proportion of early‐stage cancer in our study was similar with previous studies,^29^, ^30^ but lower than up to 70% which has been reported in some CT screening studies.^12^, ^28^, ^31^ Characteristics of the recruited population and experience of experts in diagnosis may partially explain this discrepancy. However, the high proportion of detected early‐stage cancer implies the opportunity of patients to undergo curative treatment, which may collaborate with reducing cancer mortality.

Selecting the most appropriate target candidates for lung cancer screening with LDCT is critical to maximize benefits and minimize adverse effects. Currently, entry criteria mainly based on age and smoking history, and risk prediction models are the two main methods for most screening programs to select the high‐risk population for LDCT lung cancer screening.^7^, ^32^, ^33^, ^34^, ^35^ However, other risk factors such as family history of cancer, and occupational exposure have also been considered as eligibility criteria in some study programs.^9^, ^31^ However, what best defines a high‐risk population remains unclarified. In the present study, we found that lung cancer incidence varied with baseline characteristics of participants. According to the LungSPRC criteria, the high‐risk population had a lung cancer incidence of 513.31 per 100 000 person‐years. When applying the NLST high‐risk criteria in the present study, the incidence was 877.41 per 100 000 person‐years. These results suggest more studies are needed to investigate the optimal age and smoking index of selecting high‐risk population for lung cancer screening with LDCT in China. Previous studies reported that tin miners had a higher incidence than residents.^14^ In this study, we found similar results, which indicated that it is essential to consider other risk factors except age and smoking when selecting high‐risk candidates. In the present study, participants with a negative result in the baseline screening had a lower risk of lung cancer than those with a positive result, which was similar to a previous study.^36^ Those results suggest that extending the interval between screenings in participants with a negative screening result might be needed, and methods should be found to distinguish individuals who need more or less frequent screening with LDCT. In addition, it should be a priority to call back participants with positive results for follow up when screening task is limited.

This study had some limitations. First, our study was designed with the perspective of providing public health services for the high‐risk population of lung cancer. It is not a randomized controlled trial, and we were unable to evaluate whether LDCT screening could reduce lung cancer mortality among the high‐risk population. Second, not all participants were called back for the annual screening because of limited financial support, and we may have underestimated the early detection rate of lung cancer. Third, the sample size in the present study was smaller than some studies. However, our findings do provide valuable insights for lung cancer screening with LDCT in the Chinese context.

In summary, our program provided public health services of lung cancer screening with LDCT in Gejiu, Yunnan Province. The evidence for implementation of LDCT lung cancer screening programs in China is limited. Our findings have provided insights for implementing and optimizing LDCT screening programs for the high‐risk population in China. Recruitment criteria of the high‐risk population should be refined to be more suited towards the Chinese population, and reducing the high false positive results should be prioritized.

## Disclosure

No authors report any conflict of interest.
